# Myelin oligodendrocyte glycoprotein antibody-associated optic neuritis in a COVID-19 patient: A case report

**DOI:** 10.1097/MD.0000000000025865

**Published:** 2021-05-14

**Authors:** Chio Kogure, Wataru Kikushima, Yoshiko Fukuda, Yuka Hasebe, Toshiyuki Takahashi, Takashi Shibuya, Yoichi Sakurada, Kenji Kashiwagi

**Affiliations:** a Department of Ophthalmology, University of Yamanashi, Chuo, Yamanashi; b Department of Neurology, Tohoku University Graduate School of Medicine, Sendai, Miyagi; c Department of Neurology, National Hospital Organization Yonezawa National Hospital, Yonezawa, Yamagata; d Shibuya Eye Clinic, Fujikawa, Yamanashi, Japan.

**Keywords:** case report, coronavirus disease 2019, myelin oligodendrocyte glycoprotein antibody, optic neuritis

## Abstract

**Rationale:**

Coronavirus disease 2019 (COVID-19) has spread worldwide. It involves multiple organs of infected individuals and encompasses diverse clinical manifestations. We report a case of acute optic neuritis (ON) associated with myelin oligodendrocyte glycoprotein (MOG) antibody possibly induced by COVID-19.

**Patient concerns:**

A 47-year-old man presented to our clinic with left eye pain and vision loss. Magnetic resonance imaging of the orbit revealed the bilateral high intensity of the optic nerve sheaths. He tested positive for COVID-19 by polymerase chain reaction (PCR) testing on the day of admission but he had no signs of respiratory illness. Laboratory testing revealed that MOG immunoglobulin G (MOG IgG) was positive, but other antibodies including aquaporin-4 were negative.

**Diagnosis:**

The patient was diagnosed with MOG antibody-positive acute ON possibly induced by COVID-19.

**Interventions:**

Steroid pulse therapy consisting of methylprednisolone 1 g/day for a total of 3 days, followed by an oral prednisolone taper was performed.

**Outcomes:**

His left eye pain was immediately relieved, and his decimal vision improved from 0.03 to 0.1 on the day of discharge. Outpatient follow-up 2 weeks later revealed left a decimal vision of 1.2, and a complete resolution of the left eye pain.

**Lessons:**

Our case indicated that COVID-19 might trigger an autoimmune response that leads to MOG antibody-associated ON, similar to other pathogens that were reported in the past. The treatment response to steroid pulse therapy was preferable following a typical course of MOG antibody-positive ON.

## 1 Introduction

Currently, the worldwide prevalence of the novel severe acute respiratory syndrome coronavirus 2 (SARS-CoV-2) is a great concern for most countries. SARS-CoV2, which was first reported in December 2019 by Wu et al in Wuhan, China, causes coronavirus disease 2019 (COVID-19).^[1]^ As the disease rapidly spreads worldwide, many clinical features of COVID-19, including respiratory,^[2]^ cardiac,^[3]^ neurological,^[4–6]^ and ophthalmic^[7–9]^ illness, has been reported.

Recently, myelin oligodendrocyte glycoprotein (MOG) antibodies have been reported to encompass patients with varied pathologies involving the central nervous system, including optic neuritis (ON), acute disseminated encephalomyelitis, and encephalitis.^[10–13]^

Here, we describe a case of acute ON associated with MOG antibody possibly induced by COVID-19 infection.

## 2 Case report

A 47-year-old Japanese man presented to our clinic with left eye pain and an upper visual field defect. Two days before the onset of left eye pain, his son tested positive for COVID-19 by polymerase chain reaction (PCR) testing, and he was isolated at home as a close contact. He had a medical history of right adrenal resection due to primary aldosteronism and recurrent paranasal sinusitis. He denied any history of immunological or neurological disease. He also did not have any family history of auto-immune diseases. He had no respiratory symptoms, fever, or loss of taste. He denied recent travel or contact with crowding.

Our examination revealed a decimal visual acuity of 0.2 in the left eye, with a left relative afferent pupillary defect. The average critical flicker frequency value was 42 Hz in the right eye and 20 Hz in the left eye. Eye movement was normal, although left eye pain worsened when moving the eyes. Fundus examination with pupil dilation revealed no disc edema, disc redness, retinal hemorrhage, or change in retinal vessels. Intraocular pressure was 14/14 mm Hg using a Goldmann applanation tonometer. He had symptoms of paranasal sinusitis, a stuffy nose, and flowing mucus. He tested positive for COVID-19 by nasopharyngeal PCR testing.

**Figure 1:**
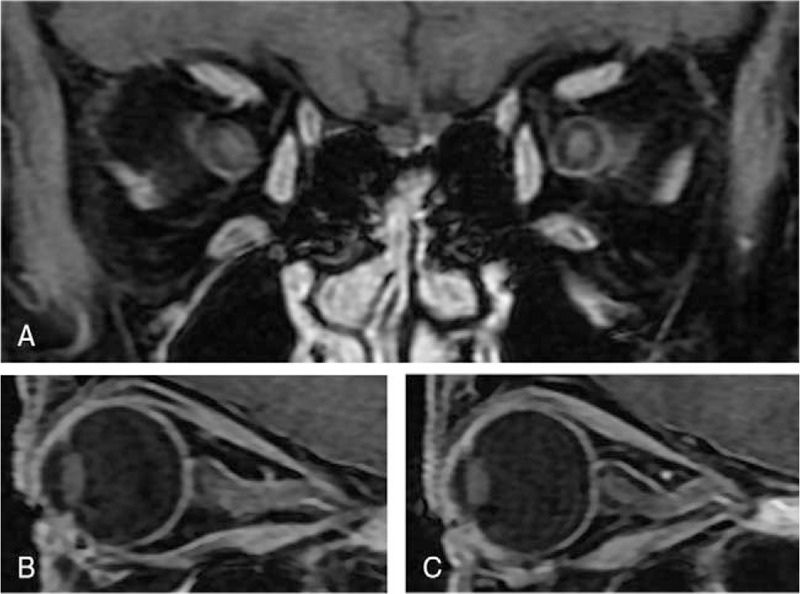
Postcontrast T1-weighted fat-suppressed magnetic resonance imaging (MRI) of the orbits. (A) Coronal MRI of the orbits reveals bilateral (but left dominant) uniform enhancement of the optic nerve. (B) Sagittal MRI of the right orbit reveals a slightly ill-defined appearance of the optic nerve and slight enhancement of optic nerve sheaths. (C) Sagittal MRI of the left orbit reveals uniform enhancement along with optic nerve sheaths.

To rule out nasal ON, we conducted magnetic resonance imaging (MRI) of the brain and orbits with gadolinium contrast. Postcontrast T1-weighted fat-suppressed MRI revealed the bilateral (but left-dominant) uniform enhancement along with optic nerve sheaths (Fig. 1). No sign of inflammation or accumulation of pus in the paranasal sinus was detected on MRI. Brain MRI revealed no sign of abnormal intensity, which is usually observed in multiple sclerosis. There was no abnormal sign on chest x-ray and computed tomography.

We also conducted blood and cerebrospinal fluid (CSF) testing. There were no abnormal blood count or biochemical profile results. The C-reactive protein level was 0.10 mg/dL and the erythrocyte sedimentation rate was 2 mm (1 mm/h). Serum test results for antinuclear antibody, QuantiFERON-TB, cytoplasmic-antineutrophil cytoplasmic antibody, perinuclear-cytoplasmic-antineutrophil cytoplasmic antibody, and aquaporin-4 were all negative. Lumbar puncture revealed no elevation of white blood cells or proteins with a normal opening pressure of 160 cmH_2_O. PCR testing of SARS-CoV-2 RNA in the CSF was negative. MOG-immunoglobulin G (MOG-IgG) testing in blood was positive with a titer of 1:128, whereas that in the CSF was negative.

**Figure 2:**
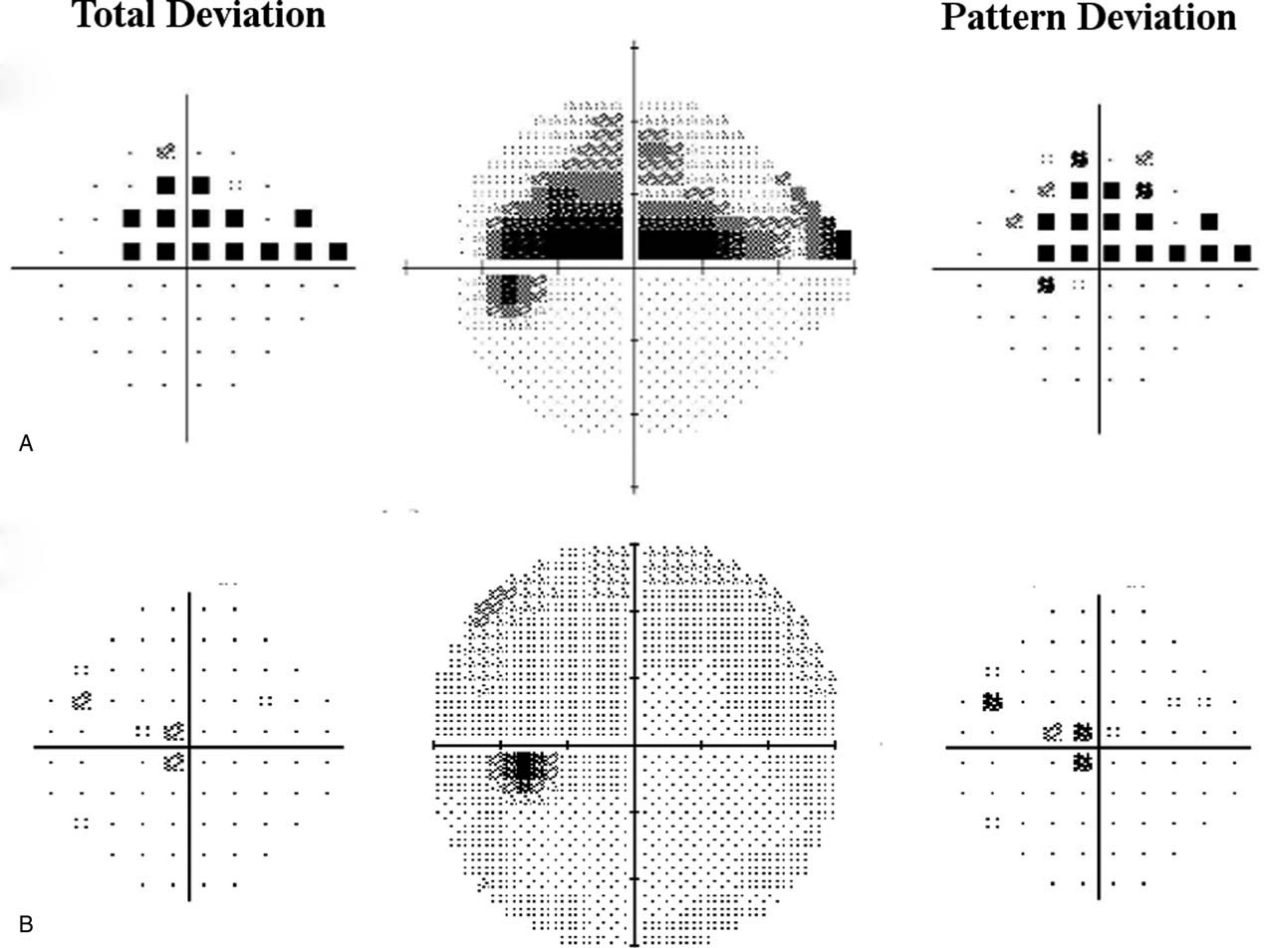
Left central visual field change in Humphrey Field Analyzer (HFA, Carl Zeiss AG, Oberkochen, Germany) before and after steroid pulse therapy. The total deviation plot is on the left and the pattern deviation plot is on the right of each gray scale. (A) Pretreatment HFA central 24-2 threshold test performed at the clinic of origin reveals a central to upper visual field defect. The visual acuity of the left eye was 0.9 on the decimal scale, which deteriorated to 0.2 at our clinic on the next day. (B) Posttreatment HFA central 30-2 threshold test performed at outpatient follow-up 2 weeks after discharge reveals an almost complete resolution of the central visual field defect. The visual acuity of the left eye improved to 1.2 on the decimal scale.

After optic and systemic examination, the patient was diagnosed with MOG antibody-positive ON possibly induced by COVID-19 and was admitted to our hospital. On the day of admission, his visual acuity in the left eye seriously deteriorated to counting finger. We had the approval of steroid pulse therapy for the patient by Institutional Review Board, then he was started on methylprednisolone 1 g/day for a total of 3 days, followed by an oral prednisolone taper. His pain with eye movement was immediately relieved after starting steroid pulse therapy, and his visual acuity subsequently improved to a level of 0.1 on the decimal scale on the day of discharge, which was the tenth day after admission. On the selfsame day, he tested negative for COVID-19 by PCR testing. The average critical flicker frequency value in the left eye also improved to 34 Hz (40 Hz in the right eye). There were no pulmonary or systemic symptoms during hospitalization. His visual acuity improved to a level of 1.2 on the decimal scale and left relative afferent pupillary defect disappeared at outpatient follow-up 2 weeks later. Figure 2 illustrates the left central visual field change in the Humphrey Field Analyzer (HFA, Carl Zeiss AG, Oberkochen, Germany) before and after steroid pulse therapy. The patient has provided informed consent to publish these features of his case, and the identity of the patient has been protected.

## 3 Discussion

Our case of a healthy Japanese man (except for paranasal sinusitis and the past history of primary aldosteronism) showed symptoms typical of acute ON. Although his vision loss and pain with eye movement appeared unilaterally, orbital contrast-enhanced MRI showed bilateral enhancement along with optic nerve sheaths. His clinical findings were in accordance with those of ON associated with MOG antibody disorder (MOGAD). In the literature, there are 2 Hispanic cases reporting ON associated with MOGAD, which were observed during COVID-19 infection. Sawalha et al reported the case of a 44-year-old Hispanic man with no remarkable past medical history.^[14]^ The patient showed acute bilateral ON with no signs of demyelination or myelitis. Another case was reported by Zhou et al. The patient was a 26-year-old Hispanic man who presented with symptoms of bilateral ON and myelitis.^[15]^ In these 2 patients, relatively mild respiratory symptoms (i.e., shortness of breath and dry cough) preceded visual impairment. Steroid pulse therapy consisting of methylprednisolone 1 g per day for a total of 5 days was administered, and immediate recovery of visual acuity and resolution of eye pain were observed in both patients. To the best of our knowledge, this is the first Asian case of ON associated with MOGAD observed in the period of COVID-19 infection. The patient in the present report was male, and had no severe pre-existing disease, similar to the 2 patients described above. The difference between our case and the previous 2 cases is that our patient showed almost unilateral (except MRI findings) ON and had no preceding respiratory symptoms. However, the patient in our report had been in close contact with an infected person in his family before he showed symptoms of ON. Thus, we consider that his symptoms of ON associated with MOGAD were possibly induced by COVID-19 infection.

There are several reports of MOG antibody-positive ON followed by prodromal infections. Nakamura et al reported the case of a 41-year-old man who presented with MOG antibody-positive bilateral ON and meningoganglionitis after herpes simplex virus infection.^[16]^ Ramanathan et al and Nakajima et al reported a case series of MOG antibody-associated ON.^[17,18]^ In the 2 studies, 37.5% to 67% of patients had prodromal infections; however, no specific pathogens were described. Therefore, the 3 cases of COVID-19 patients described above indicate that infection with SARS-CoV-2, like those with other pathogens, might trigger an autoimmune response that leads to MOG antibody-associated ON. In addition, 1 recent case report might support this hypothesis. Woodhall et al reported the case of a 39-year-old woman who experienced relapsed MOGAD following SARS-CoV-2 infection.^[19]^ She had a history of several episodes of bilateral or unilateral ON 2 to 10 years before the final relapse. At the time of the final relapse, she showed symptoms of right ON 6 days after testing positive for COVID-19 by nasopharyngeal PCR. The author suggests that the SARS-CoV-2 host response might activate MOG-IgG1 specific B-cell subsets via increased expression of pro-inflammatory cytokines. Moreover, in a study with a large sample, it was demonstrated that infection-associated relapses in MOGAD are observed in 20% of patients.^[20]^ However, it remains to be elucidated whether the symptoms of MOG antibody-positive ON of our patient were genuinely induced by COVID-19 or his ON and COVID-19 existed independently. The fact that MOG antibody testing in the CSF was negative is also confusing, however, we consider that the result of MOG antibody testing in the CSF might have been false-negative. Further studies are necessary to evaluate the association between MOGAD and infection with SARS-CoV-2.

In the literature, it was reported that, despite severe initial vision loss, the visual prognosis of patients with MOG antibody-positive ON is relatively favorable. In a recent large-scale cohort study of ON in Japan, Ishikawa et al reported that the mean logarithm of the minimal angle of resolution best-corrected visual acuity (BCVA) of 54 patients with MOG antibody-positive ON improved from 1.6 to 0 (equivalent to 0.025–1.0 on the decimal scale) after steroid pulse therapy.^[21]^ Similarly, in a multicenter retrospective cohort study, Padungkiatsagul et al reported that most subjects (51 of 77 White subjects and 52 of 70 Asian subjects) had good recovery of the BCVA (>0.5 on the decimal scale) despite their high prevalence of severe visual loss during the nadir.^[22]^ The treatment response to the steroid pulse therapy in the present report was also preferable following a typical course of MOG antibody-positive ON. We consider it encouraging that all 3 patients with MOG antibody-positive ON induced by COVID-19 had not worsened or presented with symptoms of respiratory illness during the treatment period of systemic steroids.

## 4 Conclusion

Our patient in the present report showed symptoms of acute unilateral ON. An orbital contrast-enhanced MRI showed signs of bilateral ON, and the MOG antibody was detected by laboratory testing. Other examinations, including lumbar puncture or laboratory assessment, did not support the possibility of multiple sclerosis or other autoimmune diseases. Thus, we consider that his MOG antibody-positive ON was possibly induced by COVID-19 virus infection. Similar to the case of MOGAD described in the past, steroid pulse therapy improved his symptoms. Our case indicates that MOG IgG testing could be considered in the case of COVID-19 infection preceding ON or myelitis.

## Author contributions

**Conceptualization:** Wataru Kikushima.

**Data curation:** Chio Kogure.

**Investigation:** Chio Kogure, Wataru Kikushima, Toshiyuki Takahashi.

**Resources:** Yoshiko Fukuda, Yuka Hasebe, Takashi Shibuya.

**Writing – original draft:** Chio Kogure, Wataru Kikushima.

**Writing – review & editing:** Wataru Kikushima, Yoichi Sakurada, Kenji Kashiwagi.
